# Food and nutrient security for a growing population

**DOI:** 10.1093/af/vfy014

**Published:** 2018-08-20

**Authors:** Penny K Riggs, Michael J Fields, H Russell Cross

**Affiliations:** 1 Department of Animal Science, Texas A&M University, College Station, TX; 2 Department of Animal Sciences, University of Florida, Gainesville, FL

Food and nutrient security in light of population growth projected to reach 9.8 billion over the next 30 yr (United Nations, 2017) are becoming ubiquitous concerns for professionals across agricultural sciences, health sciences, and policy fields. In addressing the challenges presented by current circumstances, this issue of *Animal Frontiers* derives from a recent International Livestock Congress (**ILC**) presented by the International Stockmen’s Educational Foundation (**ISEF**) in conjunction with the Houston Livestock Show and Rodeo. The ISEF hosts industry and world leaders and scientific experts, and supports a student educational program, in part through its annual Congress, to generate discussion and exchange of global opinions on current topics of interest and concern across the livestock industry’s entire supply chain. The most recent ILC focused on “Science-based strategies for meat in the diet and new perspectives on global trade.” This topic arose from the underlying concept that food security is one critical component of national security. Moreover, “nutrient security” must also be considered for adequate nutritional wellbeing of people in economically disadvantaged countries, as well as those that are more prosperous, in order to meet the challenges of adequate protein intake needs, conflicting nutritional advice, and activist agendas.

In this issue, David Klurfeld addresses “What is the role of meat in a healthy diet?” He describes the value of red meat as a nutrient-dense food that is a complete protein source containing all essential amino acids, iron, zinc, selenium, and B vitamins. Among animal-source foods, red meat in particular has been a target for criticism. Klurfeld describes a review process in which the World Health Organization’s International Agency for Research on Cancer ignored controlled studies and used weak epidemiological evidence in an effort to classify red meat as a carcinogen. Although questions still remain regarding dietary choices, Klurfeld notes that the most common nutrient deficiencies worldwide could be eliminated by regular consumption of a small amount of beef, emphasizing that dietary recommendations must be based on sound scientific principles.

To ensure that the U.S. Dietary Guidelines for Americans (**DGA**) continues to provide evidence-based information about dietary practices for optimal nutrition, a National Academies’ panel has made several recommendations to improve the DGA update process. Among the recommendations is a call for increased rigor in evaluating scientific evidence (NASEM, 2017). In a global assessment of nutritional guidelines and implications for the future, Magni et al. (2017) also address the importance of effectively translating nutritional guidelines for the public. Here, Graves and colleagues discuss communications strategies for delivering the nutritional benefits of meat to consumers in “Giving meat meaning: Creating value-based connections with consumers.” In particular, they offer tips for the Millennial generation who are at risk for eliminating meat from their diets.

The world market is changing, and the role of the private sector leads this transformation (Global Panel, 2018). As described by the Food and Agriculture Organization of the United Nations (FAO, 2018), growth of population and gross domestic product, along with globalization and urbanization, are driving demand for more animal-source foods, especially in low- and middle-income countries. Meeting demand requires increased productivity, structural changes to the livestock sector, and increased trade of livestock and livestock products. The remaining articles in this issue tackle aspects of this topic from different angles. The “International beef trade: A value proposition,” is described by Fields and colleagues, largely addressing U.S. beef and pork exports and exploring new opportunities. Davis and Belk provide a look at the role of technological advances in “Managing meat exports considering production technology challenges”, and discuss the necessity of technology adoption for meeting demand. In another area of technology, food animal industries are grappling with the impact of meat and dairy alternatives on these industries, as well as their implications for nutritional security. Keefe describes this issue in “#FakeMeat versus ‘real’ meat: The coming battle.”

Next, two case studies of the beef industry bring an international perspective and approach to the discussion. In “Beef cattle production system capacity considerations for improved food security: A case study in Myanmar,” Herring and colleagues propose strategies for developing livestock operations and improving beef production in that country. At the other end of the spectrum, Polkinghorne describes a strategic approach to overall industry improvement—with emphasis on product quality and marketability “From commodity, to customer, to consumer: The Australian beef industry evolution.” Finally, Daigle and Ridge wrap up this issue by addressing the importance of developing the animal science workforce and suggest that “Investing in stockpeople is an investment in animal welfare and agricultural sustainability.” They describe current barriers and offer potential solutions for developing and increasing the availability of a skilled workforce.

## About the Authors

**Dr. Penny Riggs** is an Associate Professor in the Department of Animal Science at Texas A&M University, where she chairs the Council of Principal Investigators. She also serves as the current chair of the Public Policy Committee of the American Society of Animal Science. She has broad, multidisciplinary interest in the role of animal agriculture and biotechnology for food and nutritional security. Her current research interests include genetic parameters that regulate or determine aspects of beef quality, skeletal muscle traits, and developmental processes. She completed B.S. and M.S. degrees in Biology and Cytogenetics, respectively, at Purdue University, and a Ph.D. in the interdisciplinary program in Genetics at Texas A&M University. She conducted postdoctoral research at NASA L.B. Johnson Space Center, and later, the M.D. Anderson Cancer Center, prior to returning to Texas A&M University as a faculty member.

**Dr. H. Russell Cross** has more than 45 years of research and management experience, holding numerous positions in government, academia, and the private sector. Dr. Cross is a Professor in the Department of Animal Science in the College of Agriculture and Life Sciences at Texas A&M University. He most recently served as head of the Department of Animal Science and Executive Vice President for Operations at Texas A&M University. Prior to that appointment, he served as Deputy Vice Chancellor and Associate Dean for Agriculture and Life Sciences at Texas A&M University. His service in government includes the role of Administrator of USDA’s Food Safety and Inspection Service under Presidents Bush (41) and Clinton. The USDA Food Safety and Inspection Service comprised over 10,000 employees with a budget of $600 M. At the U.S. Meat Animal Research Center in Clay Center, NE (largest animal research center in the world), Dr. Cross served as USDA’s Research Leader within the Meat Research Group. Within the private sector, Dr. Cross was the CEO and Chairman of Future Beef Operations, LLC. Furthermore, he served as Director of Food Safety Net for IDEXX Laboratories. Within National Beef Packing Co, Dr. Cross served as Executive Vice President of Food Safety/Government and Industry Affairs. In addition, he was Vice President of DuPont Food Industry Solutions.

**Dr. Michael Fields**, Professor Emeritus of Animal Sciences at the University of Florida is a second generation Florida rancher, managing the family ranching enterprise just north of Disney, and has worked closely with the owner and managers of South Florida ranches, many of whom were his classmates or his students. He has published more than 200 articles on beef cow reproduction and two books on reproductive management. To complement his work with the ranching community, he has extensive experience working with the basic biology of livestock issues in his research lab funded by the USDA, NIH, NSF, and producer organizations. This experience has placed him in an enviable position of bringing the latest technology to the efficient production of livestock. Dr. Fields has received numerous awards for his teaching, service, and research, including the University of Florida Outstanding Teacher of the Year, and Professional Award of Excellence. He served in the United States Marine Corps, received his bachelor’s and master’s degrees from the University of Florida, and his doctoral degree in reproductive physiology from Texas A&M University. Dr. Fields has been a notable voice to raise the national visibility of the beef industry in Florida.
